# The Key Lnc (RNA)s in Cardiac and Skeletal Muscle Development, Regeneration, and Disease

**DOI:** 10.3390/jcdd8080084

**Published:** 2021-07-25

**Authors:** Amanda Pinheiro, Francisco J. Naya

**Affiliations:** 1 Program in Molecular Biology, Cell Biology and Biochemistry, Boston University, Boston, MA 02215, USA; amandaep@bu.edu; 2 Department of Biology, Boston University, Boston, MA 02215, USA

**Keywords:** cardiac, skeletal, development, regeneration, disease, long noncoding RNA, epigenetics, genomic imprinting

## Abstract

Non-coding RNAs (ncRNAs) play a key role in the regulation of transcriptional and epigenetic activity in mammalian cells. Comprehensive analysis of these ncRNAs has revealed sophisticated gene regulatory mechanisms which finely tune the proper gene output required for cellular homeostasis, proliferation, and differentiation. However, this elaborate circuitry has also made it vulnerable to perturbations that often result in disease. Among the many types of ncRNAs, long non-coding RNAs (lncRNAs) appear to have the most diverse mechanisms of action including competitive binding to miRNA targets, direct binding to mRNA, interactions with transcription factors, and facilitation of epigenetic modifications. Moreover, many lncRNAs display tissue-specific expression patterns suggesting an important regulatory role in organogenesis, yet the molecular mechanisms through which these molecules regulate cardiac and skeletal muscle development remains surprisingly limited. Given the structural and metabolic similarities of cardiac and skeletal muscle, it is likely that several lncRNAs expressed in both of these tissues have conserved functions in establishing the striated muscle phenotype. As many aspects of regeneration recapitulate development, understanding the role lncRNAs play in these processes may provide novel insights to improve regenerative therapeutic interventions in cardiac and skeletal muscle diseases. This review highlights key lncRNAs that function as regulators of development, regeneration, and disease in cardiac and skeletal muscle. Finally, we highlight lncRNAs encoded by imprinted genes in striated muscle and the contributions of these loci on the regulation of gene expression.

## 1. Introduction

Only a small percentage (<2%) of the human genome, or approximately 20,000 genes, code for proteins [1,2]. The majority of the genome, however, is composed of non-coding regions including genes that code for regulatory RNAs. Numerous non-coding RNAs (ncRNAs), including microRNAs (miRNAs), long non-coding RNAs (lncRNAs), small nuclear RNAs, small nucleolar RNAs, transfer RNAs, and ribosomal RNAs play a wide variety of important roles in numerous cellular processes [3,4,5]. These ncRNAs are transcribed from genes, either individually or in clusters, and/or processed from intronic sequences of RNA transcripts.

Generally, ncRNAs such as miRNAs and lncRNAs play key roles in the regulation of gene expression [6,7]. While miRNAs regulate gene expression through their ability to degrade mRNA or inhibit translation, lncRNAs predominantly do so through their interactions with transcription factors and chromatin remodeling proteins [3,8]. Both miRNA and lncRNA genes are transcribed by RNA polymerase II, but lncRNAs are encoded by a greater number of genes (>16,000) in the mammalian genome [1,9,10].

Despite the vast number of lncRNAs minimal research has been done to evaluate these important regulatory RNAs [1,11,12]. Typically expressed at lower levels than protein-coding genes, lncRNAs are thought to be tissue-specific, suggesting a role in lineage determination [1]. The standard open reading frames (ORFs) are absent in lncRNAs but short ORFs appear to be present in some lncRNAs which encode short polypeptides [13]. While bona fide lncRNAs have no protein-coding potential, several functional peptides are transcribed from certain lncRNAs endowing them with dual regulatory functions [14,15,16]. A variety of classification systems have been proposed to categorize the types of lncRNAs by both structure and function. The diverse categories of lncRNAs have been thoroughly described in other reviews and will not be elaborated on here [17,18,19].

LncRNAs have diverse functions including competitive binding to miRNA targets, binding to mRNA directly, and interactions with key transcription factors [10,20,21,22,23]. LncRNAs are also major contributors to the epigenome through their interaction with histone-modifying enzymes to carry out chromatin remodeling. Such nuclear-localized lncRNAs can act either in cis or trans and principally interact with enhancer of zeste homolog 2 (Ezh2) subunit of the histone lysine methyltransferase PRC2 to modulate the accessibility of transcribed regions [24]. Perhaps the most dramatic example of PRC2-mediated chromatin remodeling is the imprinting of genes in development which often requires lncRNAs to modulate the activity of this histone-modifying complex.

The development of high throughput RNA sequencing has enabled the identification of new lncRNAs. To investigate these lncRNAs investigators employ various strategies including short hairpin RNA (shRNA), Adeno-associated virus (AAV), and transgenic animals to knockdown, knockout and overexpress these transcripts in cells or animal model systems. The majority of the lncRNAs discussed in this review are mammalian and are species-specific unless otherwise mentioned. Even within mammals, the low conservation across the class (mouse to human) presents challenges for translating basic science into clinical practice [25]. While most of the discovered lncRNAs are species-specific, one group identified 8953 lncRNAs which are common between humans and other species [26].

This review focuses on key lncRNAs published in the literature which are known to play a critical role in cardiac and skeletal muscle development, regeneration, and disease. In contrast to miRNAs which have received a considerable amount of attention in cardiac and skeletal muscle, much less is known about lncRNAs in these tissues. Developing a deeper knowledge of lncRNA function in striated muscle will provide a better understanding of these often overlooked players in the complex process of myogenesis. This information can ultimately be used to improve regenerative therapeutic interventions for a spectrum of muscle diseases. 

## 2. Long Non-Coding RNA in Cardiac Muscle

### 2.1. Cardiac Development

The process of cardiac development requires numerous levels of gene regulation in which lncRNAs provide an essential regulatory layer to the modulation of specification, determination, proliferation, and differentiation. Signaling molecules such as Bmp, Wnt, and Fgf, miRNAs (miR-1, miR-133, miR-206) and an array of transcription factors including *Nkx2.5*, *Mef2c*, *Gata4*, *Tbx5*, and *Hand*, constitute an elaborate and conserved gene regulatory network for early cardiac morphogenic events [27,28,29,30]. Adding to this network of key regulatory molecules, a growing number of lncRNAs such as *Miat*, *Alien*, and *H19* have been identified that are preferentially detected during embryonic development, but their functional role in cardiac development is not well understood [31].

*Braveheart* (*Bvht*) was one of the first identified lncRNAs determined to be necessary for the progression of early mesoderm towards a cardiac lineage in mice. Investigators used shRNA to knockdown *Bvht* in embryonic stem cells and determined it functions as an activator of a key gene regulatory network consisting of the cardiac transcription factors (*Mesp1*, *Gata4*, *Hand1*, *Hand2*, *Nkx2.5*, and *Tbx5*) as well as epithelial to mesenchymal transition genes (*Snai* and *Twist*). *Bvht* appeared to have similar cardiac development regulatory functions to MESP1, a gene expressed in nascent mesoderm and necessary for cardiac development, based on the in vitro rescue of the *Bvht* depleted upon MESP1 induction [32,33,34]. Additionally, *Bvht* interacts with a component of PRC2, namely SUZ12, during differentiation, thus implying its critical role in epigenetic regulation of myogenic commitment [34]. Another lncRNA in mice that interacts with histone-modifying enzymes is Fetal-lethal noncoding developmental regulatory RNA (*Fendrr*). Expressed in the nascent lateral mesoderm, this lncRNA binds to the PRC2 and Trithroax group/MLL protein complexes (TrxG/MLL) to modify chromatin structure involved in the formation of the heart and body wall. Consistent with these interactions, aberrant histone methylation patterns at promoter regions of several cardiac transcription factor genes including *GATA6* and *Nkx2.5* were detected in *Fendrr* null mutant embryos which display cardiac mesoderm defects [35]. Garcia-Padilla et al. found a dynamic isoform-specific expression and nuclear/cytoplasmic localization of *Bvhrt* and *Fendrr* during chamber formation [31]. Additionally, this group noted a change in the temporal expression of these lncRNAs which were expressed during early development but expression levels were found to peak again during adulthood [31].

Recently, an interesting regulatory relationship has been demonstrated between *Hand2* and neighboring lncRNAs. In mice, the lncRNA locus *Handsdown* (*Hdn*) is located downstream, proximal to the *Hand2* gene, and was found to be essential for embryonic development. The *Hdn* locus produces 4 major lncRNAs of which the major isoform is *Hdn* which is localized to the cytosol near the nucleus. This transcriptional activity from the *Hdn* locus is required to negatively regulate the expression level of *Hand2* via interactions with its upstream regulatory elements. Additionally, *Hdn* is haploinsufficient for heart development given that heterozygous mutants display noticeable hyperplasia of the right ventricle wall [36]. Another *Hand2* associated lncRNA, *upperhand* (*Uph*) has a unique locus that contains two enhancers that influence *Hand2* expression during cardiac development in addition to H3K27ac modifications and *Uph* is divergently expressed. It is co-expressed in a tissue-specific and temporal manner with *Hand2*. The termination of transcription of this lncRNA, but not the mature transcript, resulted in the loss of *Hand2* expression in the heart. This reveals that the expression of *Hand2* depends on enhancers located within the *Uph* locus. Furthermore, *Uph*-knockout experiments in mice demonstrated that the KO embryos failed to develop a right ventricular chamber thus resulting in embryonic lethality [37]. 

One important characteristic in development is the migration of cells. The lncRNA *BANCR* is derived from MER41, a family of primate-specific (human and non-human primates) endogenous retrovirus which are categorized as transposable elements, is exclusively produced in fetal cardiomyocytes and is co-regulated by cardiac transcription factor TBX5. *BANCR* has been shown to induce migration in cardiomyocytes in primate development and promote ventricular enlargement in vivo. Through chromatin immunoprecipitation and siRNA-mediated knockdown, *BANCR* was determined to be a downstream effector of the TEAD/YAP pathway, implicating possible connections to Hippo signaling and/or mechanotransduction [38]. Another lncRNA that contributes to the regulation of proliferation of cardiomyocytes is *uc.457*. This highly conserved lncRNA in vertebrates is differentially expressed in human patients with a ventricle septal defect and the overexpression of *uc.457* in vitro inhibited the differentiation and proliferation of cardiomyocytes [39]. An additional negative regulator of differentiation is the lncRNA *GATA6 antisense RNA 1* (*GATA6-AS1*). During the early stage of human pluripotent stem cell-induced cardiomyocyte differentiation, *GATA6-AS1* is transiently upregulated along with *GATA6*; however, knockdown of this lncRNA resulted in defective differentiation [40]. 

Also shown to be important in cardiac muscle differentiation, siRNA mediated silencing of *Ppp1r1b* in human induced pluripotent stem cell-derived cardiomyocytes (hIPSC-CM) resulted in impaired cardiomyocyte differentiation characterized by decreased expression of *Myogenin*, *MyoD1*, and *TBX5* [41]. Abnormal cardiac and skeletal muscle morphology was observed in mice with a CRISPR/Cas9 induced null allele of *Charme*. Specifically in cardiac muscle, both neonatal and adult hearts displayed dramatic cardiac enlargement characterized by increased ventricular wall and interventricular septum thickness [42]. 

### 2.2. Cardiac Muscle Regeneration 

Given the inability of the adult mammalian heart to repair damage sustained from disease or injury, there is considerable interest in understanding the mechanisms by which this organ responds to various insults and how these pathways can be targeted to promote tissue regeneration in this vital organ [43]. Due to the limited physiologic capacity for myocardial regeneration in the mammalian heart—a process observed exclusively in neonatal mice—there has been a paucity of studies describing a regulatory role for lncRNAs in this process [44]. Nevertheless, functional characterization of lncRNAs in mammalian cardiac regeneration is likely to expand in the near future based on recent reports describing numerous differentially expressed lncRNAs in this process [45,46]. Here, we focus on several lncRNAs which have been shown to directly contribute to cardiac regeneration or cardiomyocyte proliferation.

Cardiac regeneration-related lncRNA (*CAREL*) was found to be progressively upregulated in postnatal mouse hearts from postnatal days one to ten [47]. Using transgenic mice overexpressing *CAREL* in a cardiomyocyte-specific manner or intramyocardial administration of *CAREL* adenovirus, inhibited cardiomyocyte proliferation after apical resection of neonatal hearts. *CAREL* was found to act as a competing endogenous RNA (ceRNA) on miR-296, an miRNA that promotes cardiomyocyte proliferation [47]. This group identified another lncRNA, *LncDACH1*, and subsequently demonstrated its ability to modulate neonatal cardiac regeneration. Transgenic mice or adenoviral overexpression of *LncDACH1* in neonatal hearts displayed impaired cardiac regeneration after apical resection. Similar to the effects of *CAREL* lncRNA on regeneration, *LncDACH1*-mediated impairment stemmed from the inhibition of cardiomyocyte proliferation [48]. 

Since effective cardiac regeneration is largely dependent on reactivation of the cell cycle in post-mitotic cardiomyocytes, Ponnusamy et al. selected several lncRNAs from the NONCODE database and examined their temporal expression at various timepoints in mouse cardiac development and postnatally. Four lncRNAs displayed increased postnatally suggesting a role in cell cycle exit. Inhibition of one of these lncRNAs, since named cardiomyocyte proliferating regulator (*CPR*), triggered cardiomyocyte proliferation in cultured neonatal myocytes. Juvenile *CPR* knockout mice (postnatal day 14) exhibited an increased number of cardiomyocytes and expression of mitotic markers. Conversely, transgenic mice overexpressing *CPR* caused a significant decrease in cardiomyocyte proliferation in regenerating neonatal hearts post-myocardial infarction [49]. *CPR* was shown to directly interact with DNA methyltransferase 3A to promote the methylation of cysteine-phosphate-guanine (CpG) islands of minichromosome maintenance 3 promoter region, a known initiator of DNA replication and cell cycle progression [49]. 

### 2.3. Cardiac Disease

#### 2.3.1. Cardiac Hypertrophy

Several cardiac lncRNAs have been identified through differential expression analysis of cardiac disease mouse models including pressure overload providing the field with novel biomarkers and therapeutic targets for pathological remodeling. Myosin heavy chain associated RNA transcript (*Mhrt*) is one of a cluster of lncRNAs transcribed in the antisense orientation within the myosin heavy chain 7 (*Myh7*) gene. *Mhrt* is primarily localized to cardiomyocyte nuclei and its expression increases in the myocardium during postnatal maturation into adulthood. *Mhrt* was subsequently found to be downregulated in mouse hearts subjected to pressure overload. *Mhrt* binds to the helicase domain of chromatin remodeler Brg1 to form the Brg1-Hdac-Parp complex, a chromatin remodeler that is activated during pathological stress and results in the repression of *Mhrt* transcription in the heart. The restoration of *Mhrt* transcripts to pre-stress levels prevented cardiac hypertrophy and failure. This inhibition of *Mhrt* transcription is necessary for the development of cardiomyopathy and was the first lncRNA demonstrated to be cardioprotective. Additionally of note, the *Mhrt*-helicase interaction revealed a novel mechanism by which lncRNAs can modulate chromatin structure [50]. The cytoplasmic lncRNA cardiomyocyte-enriched noncoding transcript (*Caren*) was recently identified in a gene-trapping screen and shown to be increased in pressure overload and angiotensin II-induced models of cardiac hypertrophy. Interestingly, it was shown to decrease translation of histidine triad nucleotide-binding protein 1 (Hint1) mRNA. *Caren* also functions in a cardioprotective manner through the suppression of Hint1, an activator of the ataxia telangiectasia mutated (ATM)-DNA damage pathway—which itself is activated in response to cardiac stress. Moreover, *Caren* maintains mitochondrial function by enhancing mitochondrial biogenesis [51]. Another lncRNA shown to be important in pressure overload models of cardiac hypertrophy is *H19*. Viereck et al. found *H19* to be downregulated during pathological cardiac hypertrophy in mice, pigs, human engineered heart tissue (hEHT) subjected to afterload, and in human tissue from various cardiac diseases including hypertrophic cardiomyopathy [52]. To determine its function in vivo, *H19* knockout mice were generated and found to enhance cardiac hypertrophy in mice whereas AAV9-mediated overexpression blunted this response to pressure overload.

Understanding how the heart becomes fibrotic in chronic heart disease—resulting in increased myocardial stiffness—is of utmost importance. Piccoli et al. performed transcriptomic profiling on cardiac fibroblasts isolated from mouse hearts subjected to pressure overload to identify dysregulated lncRNAs as potential regulators of fibrosis. The lncRNA *Meg3* was among several differentially expressed lncRNA transcripts, was found to be enriched in cardiac fibroblasts and its expression was downregulated during late-stage cardiac remodeling. GapmeR mediated silencing of *Meg3* in the heart of pressure overloaded mice reduced fibrosis and cardiomyocyte hypertrophy. The reduction in fibrosis likely stemmed from reduced expression of MMP-2, a matrix metalloproteinase involved in fibrotic remodeling of the heart. Furthermore, *Meg3* was found to interact with p53 to regulate expression of *Mmp-2*, a known target gene of this tumor suppressor in cancer cells [53]. 

#### 2.3.2. Myocardial Infarction

One of the first lncRNAs known to be implicated in any form of cardiac disease was myocardial infarction (MI)-associated transcript (*MIAT*). Ishii et al. discovered single nucleotide polymorphisms in the *MIAT* gene in humans which increased susceptibility to MIs [54]. Several other lncRNAs have been evaluated as potential biomarkers for MIs in humans including *LIPCAR*, cyclin-dependent kinase inhibitor 2B antisense RNA 1 (*ANRIL*), potassium voltage-gated channel, KQT-like subfamily, member 1 opposite strand/antisense transcript 1 (*KCNQ1OT1*), and metastasis-associated lung adenocarcinoma transcript 1 (*MALAT1*) [55,56]. One study found that peripheral blood levels of both *ANRIL* and *KCNQ10T1* were able to predict left ventricular dysfunction at 4-months post-myocardial infarction [56]. Another group analyzed plasma levels of the mitochondrial lncRNA *LIPCAR* following a myocardial infarct. It was found that *LIPCAR* levels are downregulated and then subsequently the levels increase which corresponds to ongoing cardiac remodeling. High levels of *LIPCAR* were associated with a greater risk of cardiovascular death [55]. The lncRNAs proposed as biomarkers require further evaluation to determine a specific mechanism of action in cardiac muscle.

In mice, another mitochondrial-associated lncRNA named cardiac apoptosis-related lncRNA (*CARL*) has been shown to suppress mitochondrial fission and apoptosis. Through targeting miR-539 and a prohibitin complex subunit (PHB2), *CARL* acts as a miR-539 sponge to help regulate PHB2 expression and modulate cardiac remodeling post-MI [57]. *LncDACH1*, previously described in neonatal cardiac regeneration also modulates cardiomyocyte proliferation in adult hearts subjected to myocardial infarction. Mechanistically, *LncDACH1* acts as an inhibitor of protein phosphatase 1 catalytic subunit alpha (PP1A) to influence the dephosphorylation and nuclear localization of YAP1 thus inhibiting cardiomyocyte proliferation [48]. Another negative regulator of cardiomyocyte proliferation is the cardiomyocyte regeneration-related lncRNA (*CRLL*). The knockdown of *CRLL* resulted in improved cardiac function and decreased fibrosis post-myocardial infarction in adult rats [58]. *CPR* lncRNA was also examined in adult mice subjected to MI. Consistent with its control of cardiomyocyte proliferation, *CPR* knockout mice displayed increased proliferation and improved cardiac function [49]. Commonly associated with MI, the effects of hypoxia were examined in a rat cardiomyocyte-like cell line, H9c2, which resulted in the upregulation of *Meg3*. This upregulation correlated with changes to H9c2 viability and migration [59]. 

Most recently, *CARDINAL*, a lncRNA transcribed from a neighboring region upstream of the *Myocardin* gene, was identified in a bioinformatics screen and is a cardiac-enriched, chromatin-associated lncRNA. Its deletion in mice exacerbated pathological remodeling, i.e. decreased contractility, following MI. Functionally, *CARDINAL* interacts with the SRF/TCF transcription factor complex to inhibit promitogenic genes in the heart [60]. A summary of lncRNAs that function in cardiac muscle or the heart is provided in Table 1.

## 3. Long Non-Coding RNA in Skeletal Muscle

### 3.1. Skeletal Muscle Development 

This section is focused on the fundamental aspects of myogenesis in embryonic development and the lncRNAs that help regulate this process. The mesodermal layer formed during gastrulation goes through a transition from epithelial cells to mesenchymal cells and can be divided into the paraxial, intermediate, and lateral mesoderm [61]. This layer gives rise to skeletal muscle along with cardiac muscle, most smooth muscle, cartilage, blood vessels, and bone marrow. The mesoderm is segmented into clusters of cells called somites which give rise to the vertebral column, trunk muscles, and dermis of the skin. During myogenesis, a subset of precursors, satellite cells, also originates from these multipotent mesodermal progenitor cells [62]. The fusion of committed mesodermal progenitor cells, or myoblasts, subsequently produces multi-nucleated myofibers. 

Muscle specification and differentiation are initiated by the Pax family of transcription factors (Pax3 and Pax7) and myogenic regulatory factors (MRFs): MyoD, Myf5, myogenin, Mrf4, and the MEF2 family of transcription factors [63,64,65,66,67]. Much has been learned about the transcriptional cascade in myogenesis and comprehensively examined in numerous excellent reviews [63,64,65,68]. These factors will only be described in terms of their potential connection to lncRNAs in the regulation of muscle gene programs.

In mice, the antisense divergent lncRNA *Evx1as* plays a role in the regulation of mesodermal differentiation through the facilitation of EVX1 transcription. This lncRNA *Evx1as* binds to regulatory sites on chromatin to promote an active chromatin state and interacts with the transcriptional coactivator Mediator. Chromatin immunoprecipitation (ChIP) revealed both MED1 and MED12 components of Mediator bind to the promoter and enhancer of *Evx1as*/EVX1 in mouse embryonic stem cells. *Evx1as* acts in *cis* to promote the transcription of EVX1 and upstream of EVX1 to mediate mesodermal linage differentiation [69]. Another divergent lncRNA, *yylncT* is transcribed from the *BRACHYURY* (*T*) locus has been shown to have an important role in mesodermal commitment. Frank et al. determined that *yylncT* binds to the de novo methyltransferase DNMT3B and the transcription of this lncRNA is required for activation of the T locus which is a known mesodermal specifier. Upon depletion of *yylncT*, human embryonic stem cells did not differentiate into mesoderm but the differentiation of endoderm and ectoderm remained unchanged [70].

### 3.2. Skeletal Muscle Differentiation and Regeneration 

After skeletal muscle injury, the highly coordinated process of regenerating damaged tissue involves numerous muscle and non-muscle cell types and is initiated with an inflammatory phase. This phase is characterized by the infiltration of macrophages, eosinophils, Tregs, neutrophils, and relevant cytokines [64,71]. Fibroadipogenic progenitors (FAPs) are interstitially located between muscle fibers and contribute to the stem cell niche. These non-myogenic cells play an important role in the regeneration process.

The formation of new muscle fibers, however, requires the activation and differentiation of muscle stem cells which are located between the basal lamina and myofiber sarcolemma. Quiescent satellite cells express the paired box transcription factor Pax7 [66]. Signals from the site of injury cause these cells to exit the quiescent phase and proliferate. These activated satellite cells, ultimately fuse to form multinucleated myotubes following a process similar to development. 

In addition to Pax7 and the MRFs, many lncRNAs contribute to the regulation of myogenesis in regeneration. These lncRNAs play key roles in mediating histone modifications and interactions with other RNAs to modulate myocyte function and behavior such as interactions with non-muscle cells in the regenerating microenvironment. Many of the identified lncRNAs involved in skeletal muscle regeneration act to promote myogenic differentiation, including *LncMyoD*, *linc-MD1*, *lnc-YY1*, *lnc-mg*, *SRA*, *MUNC*, *Malat1*, *Dum*, *H19*, and *Meg3*. While other lncRNAs have been shown to negatively regulate myogenesis including *m½-sbsRNA*, *YAM*, *lnc-31*, *Syisl*, and *Sirt1 AS.*

#### 3.2.1. Positive Regulators 

*LncMyoD*, transcribed 30 kb upstream of the *MyoD* gene in mice, is a direct downstream target of MyoD and serves as a key regulator of myoblast cell-cycle exit and myogenesis. This lncRNA is strongly upregulated during the differentiation process from myoblasts to myotubes. *LncMyoD* acts by blocking IGF2-mRNA-binding protein 2 (IMP2) mediated shuttling of particular mRNAs thereby affecting genes that rely on IMP for efficient translation [72,73]. The human ortholog *hLncMyoD* was determined to have conserved functions, making it one of the few functionally conserved lncRNAs in humans and mice [73]. 

Also involved in skeletal muscle differentiation in mice, *linc-MD1* acts as a ceRNA in the cytoplasm as it sponges *miR-133* and *miR-135*, which in turn upregulates the expression of the muscle-specific gene transcription factors, Mastermind-like1 (MAML1) and Myocyte-specific enhancer factor 2C (MEF2C) respectively [74]. This triple level of control confers strong regulation for the late stages of muscle differentiation. Under growth conditions in mouse myoblasts, *linc-MD1* was not present until conditions switched to differentiation conditions suggesting a regulatory role in the timing of differentiation [74]. Additionally, the RNA binding protein Human antigen R (HuR) is repressed by *miR-133* [75]. Thus, *linc-MD1* can mitigate the effects of *miR-133* on HuR through the binding of *linc-MD1* to HuR [76]. 

In mice, myogenesis-associated lncRNA (*lnc-mg*) also promotes myogenesis through its action as a ceRNA and it is required for myogenic differentiation. *Lnc-mg* knockdown in mice resulted in impaired differentiation and muscle atrophy in vivo [77]. Acting as a sponge, *lnc-mg* blocks *miR-125b* to regulate the protein levels of insulin-like growth factor 2 (Igf2) which is an important regulator of myogenesis both in vitro and in vivo [77]. Given the nodal function of MyoD in the control of myogenesis, there are several lncRNAs that are regulated by this MRF or are involved in regulating its activity. The lncRNA *linc-YY1* binds the transcription factor Yin Yang 1(YY1) allowing for YY1/PRC2 eviction from the promoters of established myogenic markers such as *Myogenin*, *MyHC*, *Tnni2*, and *α-Actin*. This eviction enables MyoD binding and subsequent gene activation. Additionally, *linc-YY1* was found to be functionally conserved between humans and mice [78]. In muscle, there exists a lncRNA transcribed from one of the upstream enhancers that regulate MyoD expression. MyoD upstream non-coding (*MUNC*/DRReRNA) overlaps with the *cis*-acting distal regulatory region (DRR) of MyoD in mice [79]. Short interfering RNA-mediated knockdown of MUNC both in vitro and in vivo resulted in impaired differentiation and regeneration, respectively. Mechanistically, there was decreased association of MyoD to the DRR enhancer and myogenin promoter demonstrating this lncRNA’s role in the regulation of MyoD function [79]. The lncRNA *Dum* [Developmental pluripotency-associated 2 (Dppa2) Upstream binding muscle] is another lncRNA involving MyoD. In this instance, upon mouse myoblast differentiation, *Dum* is induced by MyoD to promote myogenesis. It utilizes its nearby location to silence the neighboring gene *Duppa* and contributes to the recruitment of DNA methyltransferases (Dnmt1, Dnmt3a, Dnmt3b) [80]. Also pertinent to skeletal muscle differentiation, *Malat1* is upregulated in proliferating myoblast and contributes to changes in histone methylation through a mechanism discussed in further detail later in the review [81,82].

Finally, there are at least two lncRNAs encoded by imprinted genes (which are discussed in a separate section) that also function as a positive regulator of myogenesis. Short interfering RNA-mediated knockdown of the lncRNA *H19* impaired differentiation of C2C12 myoblasts and human satellite cells [83]. Curiously, knockout of *H19* in mice resulted in skeletal muscle hyperplasia and hypertrophy suggesting a negative effect on myogenesis [84]. However, the number of satellite cells was reduced indicating a positive function in proliferation and expansion of these muscle precursors. Recently, our group has shown that lncRNA *Meg3* (*Gtl2* in mice) regulates epithelial to mesenchymal transition (EMT) in murine myoblast differentiation and skeletal muscle regeneration through its regulation of genes in the TGFβ pathway via modulation of PRC2 enrichment at these loci. The knockdown of *Meg3* resulted in altered cell state of myoblasts, impaired myotube formation in vitro, and impaired regeneration in vivo. Additionally, sh*Meg3* knockdown in injured muscle, stimulated abnormal proliferation and expansion of mesenchymal stromal cells (fibroadipogenic progenitors) indicating a role of *Meg3* in non-muscle cells within the regenerative microenvironment [85]. 

#### 3.2.2. Negative Regulators 

Alternatively, some of the identified lncRNAs have been found to repress the myogenic process including *m½-sbsRNA*, *YAM*, *lnc-31*, and *Sirt1 AS*. Some mRNAs are subject to Staufen 1(Stau1)-mediated mRNA decay (SMD) which involves base pairing between Alu elements containing lncRNAs and the mRNA 3′UTR containing short interspersed element (SINE). Degradation of mRNAs occurs via SMD in TRAF6 mRNAs when they are targeted by *m½-sbsRNA(B2)* lncRNAs and this action works to promote cell activation in mice [71,86]. In mice, a YY1 associated lincRNA (*Yam-1*) is regulated by YY1 and activates in *cis miR-715* expression which is known to target Wnt7b transcripts to inhibit myogenic differentiation. Thus, *Yam-1* serves to inhibit myogenesis and thus, is downregulated upon differentiation [87].

Shown to be highly expressed in proliferating murine myoblasts, *lnc-31* acts to promote the proliferation of myoblasts and inhibits differentiation through controlling the expression of regulatory cell cycle genes. Therefore, *lnc-31* is strongly downregulated upon differentiation. Although the primary transcript is exclusively nuclear, the mature *lnc-31* is localized to the cytoplasm where it positively regulates translation of ROCK1, a Rho-associated kinase that inhibits myogenesis [88]. Characterization of *lnc-31* revealed a genomic overlap with the *miR-31* coding region. Uniquely it is theorized that both the miRNA and the lncRNA are independently produced from the same primary transcript [89]. Sirtuin type 1 Antisense (*Sirt1 AS*) lncRNA was found to interact with *Sirt1* mRNA to form an RNA duplex by competing with *miR-34a* to inhibit myogenesis. Through in vitro experiments using C2C12 myoblasts, Wang et al. showed *Sirt1 AS* lncRNA promotes myoblast proliferation and inhibits myoblast differentiation [90]. Another key repressor of C2C12 myoblast differentiation is *Syisl* lncRNA which recruits the Ezh2 protein to the promoters of p21 (cell-cycle inhibitor gene), myogenin, muscle creatine kinase, and myosin heavy chain 4, resulting in the H3K27me3, and the silencing of target genes. Consistent with in vitro studies, the results of *Syisl* knockout in mice demonstrated the functional role of *Syisl* to promote myoblast proliferation, increase myoblast fusion and inhibit differentiation [91].

*Chronos*, an age-related lncRNA in mice, is negatively regulated by Akt signaling which plays an important role in the PI3K/Akt/mTOR pathway for muscle hypertrophy. Mechanistically, *Chronos* plays a repressive role in Bmp7 transcription through the recruitment of Ezh2, thus functioning to inhibit hypertrophic growth [92].

### 3.3. Skeletal Muscle Disease

Further study of skeletal muscle lncRNAs in vivo may facilitate a deeper understanding of how abnormalities in epigenetics and gene regulation contribute to a disease phenotype. Interestingly, a subset of lncRNAs dysregulated in muscular dystrophy has revealed a non-genomic function for these regulatory molecules.

Three of the aforementioned lncRNAs that function in skeletal myogenesis have been examined in models of muscular dystrophy. Downregulation of *linc-MD1* was observed in DMD and dystrophin-deficient *mdx* mice, and its overexpression partially rescued myogenic differentiation in dystrophic muscle cells [74]. Additionally found to be implicated in dystrophin-deficient muscular dystrophy, *H19* has been shown to function in a non-genomic manner by associating and stabilizing dystrophin. *H19* was found to interact directly with dystrophin and overexpression of this lncRNA attenuated the increased ubiquitin-mediated degradation of dystrophin caused by a subset of dystrophin mutations [93]. Consistent with its function as a negative regulator of myogenesis, *lnc-31* transcripts typically are abundant during the proliferation of myoblasts and are downregulated upon differentiation. However, in primary myoblasts from patients with DMD, the downregulation of *lnc-31* is less pronounced, consistent with the delay in differentiation typical in dystrophic muscle [89]. In human patients with the most common myopathy, Facioscapulohumeral muscular dystrophy (FSHD), the D4Z4 repeat deletion is associated with reduced Polycomb silencing, contributing to the disease phenotype. The lncRNA *DBE-T* facilitates the de-repression of the 4q35 genes through direct binding of Trithorax group protein, Ash1L [94]. 

### 3.4. LncRNAs That Function as ncRNAs and Micropeptides

There is increasing literature regarding lncRNAs that have been found to code for micropeptides or “hidden peptides,” typically under 100 amino acids in length [14,95]. Some lncRNAs have dual functionality in that the RNA transcript can be translated into a micropeptide and/or maintain a coding-independent function as a lncRNA. The steroid receptor RNA activator (SRA) gene found in humans and mice produces a functional lncRNA that exists in ribonucleoprotein complexes and contributes to the nuclear receptor-mediated regulation of gene expression [96]. The translated protein product of SRA, also known as SRA protein (SRAP), works in conjunction with RNA helicases p68/p72 as co-activators of MyoD to facilitate skeletal muscle differentiation [97]. During muscle myogenic differentiation of human satellite cells, the ratio between the lncRNA and coding SRA isoform increased [98].

In addition to sponging miRNAs, circRNAs function as a medium for protein interactions and may compete with linear RNA production thus regulating the accumulation of the mRNA transcript. One such circRNA is circ-ZNF609, which is expressed in human and murine myoblasts. It has been found to be differentially expressed during the stages of myogenesis and is indicated in the control of myoblast proliferation. Uniquely, it is highly methylated and has an open reading frame that can be translated into a protein. Known to be lacking the zinc-finger domains present in the protein product from the linear transcript, the exact function of this protein is still to be determined. There is lower translation efficiency compared to linear transcripts associated with circRNAs in general because of their cap-independent translation. Therefore, there is a requirement for a splicing-dependent process for efficient translation. The existence of an internal ribosome entry site is also hypothesized in this circRNA. Circ-ZNF609 is also implicated to be more abundant during differentiation in vitro for cells with DMD perhaps contributing to their slower progression into differentiation [99].

The LINC00961 codes for a micropeptide, SPAR, is 90 amino acids long. SPAR might play a crucial role in mTOR-mediated regeneration as SPAR inactivation promotes muscle regeneration through increased mTORC1 activation [95]. Found in both humans and mice, myoregulin is another micropeptide that is encoded in a lncRNA. This transmembrane alpha-helix structured micropeptide interacts with SERCA to impede calcium uptake back into the sarcoplasmic reticulum [14]. An lncRNA-derived micropeptide 34 amino acids in length encoded by the mouse genome was termed dwarf open reading frame (DWORF). DWORF has been found to localize to the sarcoplasmic membrane and displaces SERCA inhibitors thus enhancing SERCA activity in muscles [100]. Thus, these lncRNA-derived micropeptides have functional physiologically relevant activity in muscles that must not be overlooked. A summary of lncRNAs that function in skeletal muscle is provided in Table 2.

## 4. LncRNAs Co-Expressed in Cardiac and Skeletal Muscle

**Figure 1:**
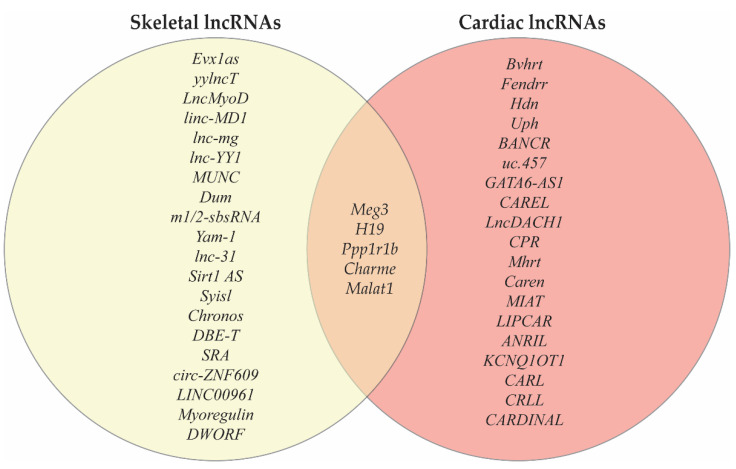
The Venn diagram includes key lncRNAs identified to play an important role in skeletal and/or cardiac muscle. The lncRNAs on the left, inside the yellow circle, are not necessarily only found in skeletal muscle but play an important role in skeletal muscle. The lncRNAs implicated in skeletal muscle development include *Evx1as* and *yylncT.* In contrast, the lncRNAs *LncMyoD*, *linc-MD1*, *lnc-mg*, *lnc-YY1*, *MUNC*, *Dum*, *m½-sbsRNA*, *Yam-1*, *lnc-31*, *Sirt1 AS*, *Syisl*, *Chronos*, and *DBE-T*, are primarily involved in skeletal muscle regeneration and disease. The skeletal muscle micropeptides include SRA, circ-ZNF609, LINC00961, Myoregulin, and DWORF. Similarly, the lncRNAs on the right, inside the red circle are not all exclusively expressed in cardiac tissue but have been demonstrated to play a role in the heart. The lncRNAs implicated in cardiac development include *Bvhrt*, *Fendrr*, *Hdn*, *Uph*, *BANCR*, *uc.457*, *and GATA6-AS1*; while the lncRNAs primarily involved in cardiac regeneration and disease are: *LncDACH1*, *CAREL*, *MIAT*, *Mhrt*, *Caren*, *LIPCAR*, *ANRIL*, *KCNQ1OT1*, *CARL*, *CRP*, *CRLL*, *and CARDINAL.* Notably, there are a number of lncRNAs that have demonstrated roles in both cardiac and skeletal muscle including *Meg3*, *H19*, *Ppp1r1b*, *Charme*, and *Malat1.*

While many lncRNAs are predominately expressed in a tissue-specific manner, there are a number of lncRNAs that have been identified to be expressed in both cardiac and skeletal muscle (Figure 1). Given the common mesodermal origin of these striated muscle cell types and the shared expression of numerous structural and metabolic genes the existence of lncRNAs that function as central regulators of gene expression in myogenesis is a strong possibility. Implicated in both cardiac and skeletal muscle development, *Ppp1r1b*-lncRNA was found to be required for normal myogenesis in a C2C12 cell model. Kong and colleagues showed that *Ppp1r1b*-lncRNA regulates myocyte differentiation by negatively modulating H3K27me3 of myogenic transcription factor genes through competitive binding for PRC2 binding with chromatin [41]. Another lncRNA found in both skeletal and cardiac muscle is Chromatin architect of muscle expression (*Charme*) which has conservation between mice and humans. *Charme* expression was localized to the nucleus primarily associated with chromatin. The knockdown of *Charme* resulted in the downregulation of key myogenic genes and defects in myoblast fusion [42].

First implicated in the metastasis of lung tumors, *Malat1* is a regulator of cell growth and proliferation in skeletal muscle. As a downstream target of myostatin, it contributes to the regulation of myogenesis [81]. *Malat1* was also found to be upregulated in C2C12 cells and primary human myoblasts during differentiation. Highly expressed in proliferating cells, *Malat1* facilitates the recruitment of the histone methyltransferase Suv39h1 to the MyoD-binding loci which leads to the stabilization of the Suv39h1/HDAC1/HP1β complex. This complex allows for the trimethylation of Histone 3 lysine 9 (H3K9me3) resulting in the repression of the target gene [82]. In cardiac muscle, *Malat1* has been evaluated as a potential biomarker for identification of myocardial infarctions [56]. The genes *H19*, *Meg3*, and *cdknc* are encoded by imprinted genes and expressed in both cardiac and skeletal muscle. For this reason, these genes will be discussed in detail in the following section. 

## 5. LncRNAs and Genomic Imprinting

Epigenetic modifications, such as DNA methylation and histone modifications, play an important role in the regulation of gene expression. Genomic imprinting is the result of differential DNA methylation patterns or posttranslational histone modifications between the paternal and maternal allele which allows for the inheritance of certain traits to occur in a parent-of-origin specific manner [101,102]. Imprinted genes are highly expressed during the prenatal developmental stage and expression decreases after birth. One proposed idea is that imprinting evolved in mammals because progeny are nourished directly from maternal tissues and as a result imprinting plays a role in the control of resources and helps to fine tune the amount of energy invested in a given offspring. Therefore, genes that are expressed during embryonic development can impact the amount of nutrients the offspring obtains from the mother [103]. There are approximately 100 imprinted genes that have been identified in mammals and many of which are positioned in 1Mb clusters that typically contain at least one ncRNA. Genes that are imprinted are essential for prenatal growth control, the establishment of different lineages, normal brain function, and postnatal energy homeostasis [104]. Given the function of imprinted genes including those encoding ncRNAs in the postnatal brain, do imprinted ncRNA loci play a role in other postnatal tissues such as cardiac and skeletal muscle?

There are a few imprinted genes/loci that are notable in striated muscle including *H19*, *Dlk1-Dio3*, and cyclin-dependent kinase inhibitor (*Cdkn1c*). The *H19* gene is highly expressed during murine embryonic development and is repressed after birth except in skeletal muscle. *H19* was initially identified in a genetic screen for muscle differentiation along with MyoD and was at the time referred to as MyoH [105]. The imprinting of the *H19* gene occurs on the paternal allele and thus is only expressed on the maternal allele. This *H19* gene codes for an lncRNA and miRNAs and has been found to be upregulated in myoblast differentiation and skeletal muscle regeneration [83]. Regarding imprinting, regenerating skeletal muscle from *H19*-deficient mice revealed an upregulation of several imprinted genes suggesting regulatory crosstalk between imprinted loci [84]. In contrast, *H19* is downregulated in cardiac development in postnatal mice and also downregulated in human and murine cardiomyocytes following a hypertrophic stimulus. As described in a preceding section in this review, *H19* also plays a role in stabilizing Dystrophin [93].

The *Dlk1-Dio3* locus is a mega-cluster of coding and ncRNA genes spanning over 200 kilobases-comprised of three protein-coding genes (*Dlk1*, *Rtl1/Peg11*, *Dio3*), and numerous lncRNAs, snoRNAs, and miRNAs. Within this cluster, the protein-coding genes are exclusively expressed from the paternal allele whereas the lncRNAs and miRNAs are encoded from the maternal allele [106]. Importantly, MEF2 is required for the expression of miRNAs from the *Dlk1-Dio3* locus in muscle and is known to play a role in differentiation [107,108]. In between *Dlk1* and *Dio3* is an ncRNA mega cluster that produces several notable ncRNAs including *Gtl2* (human *Meg3*), *Anti-Rtl1 miRNAs*, *and Rian* [109,110,111]. Given the size of the ncRNA region, the in vivo role of the entire cluster has not been determined. However, mutations have been generated in mice to investigate the importance of the differentially methylated regions (DMRs) in regulating the imprinting of this locus [112,113]. Of note, one particular mutation in mice—a deletion of the proximal promoter and first five exons of *Gtl2*—resulted in abnormal skeletal muscle morphology in perinatal mutant mice. This deletion caused the aberrant methylation pattern in the upstream intergenic DMR resulting in activation of silenced paternal genes and downregulation of all materal lncRNAs in the locus. These results demonstrate importance of proper imprinting at this locus and a collective role of maternally expressed ncRNAs in skeletal muscle development [113]. 

The *cdknc* gene is imprinted and only expressed on the maternal allele. It is highly expressed in skeletal muscle and other somatic tissues during development including the heart, kidney, and lung. The normal expression of *cdkn1c* is associated with cessation of cell cycle thus may result in defects in organ size when cdkn1c levels are dysregulated. In skeletal muscle, it is implicated in the control of proliferation and differentiation of myoblasts [114].

At this time, it is unknown to what extent imprinting at these ncRNA (and other) loci is necessary for proper cardiac and skeletal muscle homeostasis. Based on the proposed function of imprinted genes in regulating nutritional resources in developing mammalian embryos it is plausible that the lnRNAs encoded by such genes are required for modulating energy production in cardiac and skeletal muscle cells under certain physiological and pathological contexts. Along these lines, imprinting would presumably impact lncRNA levels transcribed at these loci suggesting that dosage is a critical determinant of their function in bioenergetics.

## 6. Conclusions

**Figure 2:**
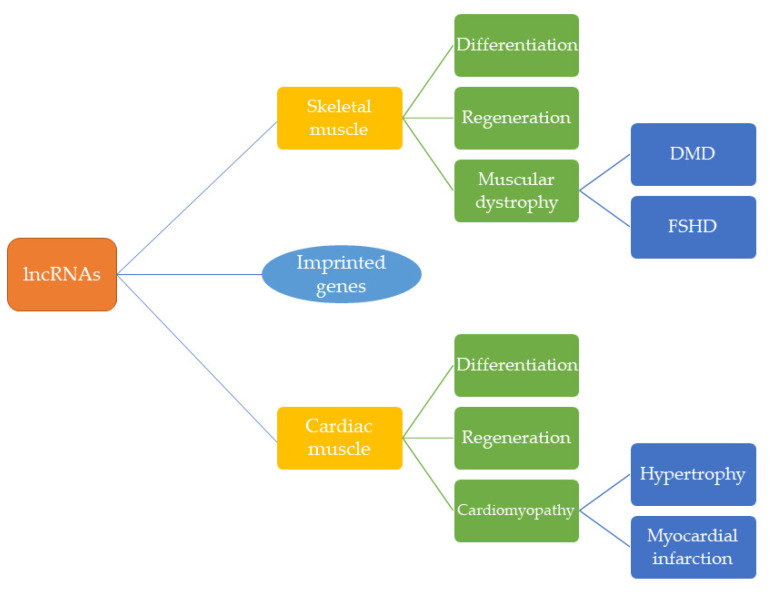
Summary of lncRNA function in cardiac and skeletal muscle. Within skeletal muscle, lncRNAs are involved in regulating differentiation in development, serve as positive and negative regulators of regeneration, and have been shown to contribute to muscular dystrophy phenotypes in both Duchenne muscular dystrophy (DMD) and Facioscapulohumeral muscular dystrophy (FSDH). In cardiac muscle, lncRNAs tightly regulate myocardial development, may facilitate neonatal cardiac regeneration, and mediate pathogenic mechanisms or serve as biomarkers in myocardial infarction and cardiac hypertrophy. Some lncRNAs expressed in cardiac and skeletal muscle are encoded by imprinted genes but the importance of this epigenetic regulation for proper homeostasis in these striated cells is not known.

As the number of investigations into lncRNA function in cardiac and skeletal muscle has increased, it has become clear that these regulatory RNA molecules play a key role in the formation of mature contractile cells from mesodermal tissue. Many of the lncRNAs described in this review regulate important signaling pathways—often through direct modulation of transcriptional or epigenetic activity—in the specification, differentiation, and regeneration of striated muscle cells (Table 1 and Table 2). In disease, lncRNAs are dysregulated and, consequently, have the potential to serve as biomarkers in pathogenesis such as cardiac hypertrophy, myocardial infarction, and muscular dystrophy (Figure 2). Curiously, a number of lncRNAs are encoded by imprinted loci thereby following the parental-specific pattern of expression of these epigenetically regulated genes. Thus, the dosage of these regulatory RNAs may need to be finely tuned for proper control of gene expression. Moreover, while many imprinted genes harboring lncRNAs and other ncRNAs are downregulated after birth, their expression is reactivated in disease. Thus, a better understanding of the regulation of imprinted loci harboring lncRNAs such as *H19* and *Meg3* may yield insight into not only the role of these ncRNA molecules but the importance of genomic imprinting in muscle development and homeostasis.

Given the structural complexity of lncRNAs, the relatively recent identification of many of the lncRNAs discussed in this review begs for a more comprehensive analysis of their mechanism of action in muscle beyond their expected interactions with chromatin modifiers and miRNAs. High throughput screens to identify novel interacting partners—proteins or RNAs—could provide insight into unrealized functions such as the fascinating regulatory relationship between *H19* and dystrophin [93]. Although progress has been made to understand the role of lncRNAs in striated muscle, much of the work has focused on in vitro studies. Future studies examining the in vivo function of these lncRNAs are warranted. Furthermore, low conservation across species requires more investigations of human orthologs and human-specific lncRNAs for clinical relevance and as biomarkers of disease. In conclusion, skeletal and cardiac muscle include lncRNAs that promote myogenesis and others that suppress it, and the balance between the two contributes to the success of this process. Understanding the dynamic interplay between these two functional categories of lncRNAs in striated muscle will provide a more complete understanding of not only the genetic circuitry in development and regeneration but how the scales are tipped in disease. 

## Figures and Tables

**Table 1:** Summary of key lncRNAs found in cardiac muscle.

Name	Species	Function	Mechanism	Citation
Hdn *	m	Cardiac development; Negative regulation of *Hand2*	Transcriptional regulation of *Hand2*	[36]
Uph *	m	Ventricle development; *Hand2* activation	Transcriptional regulation of *Hand2*	[37]
Bvht *	m	Activation of cardiovascular gene network; Epigenetic regulation	SUZ12 interaction	[34]
Fendrr *	m	Chamber development	Binds to PRC2 and TrxG/MLL complexes	[31,35]
BANCR *	p	Cardiomyocyte migration	Downstream effector of TEAD/YAP	[38]
uc.457 *^	m	Cardiomyocyte proliferation	Transcription factor interactions	[39]
GATA6-AS1 *	h	Cardiomyocyte differentiation	Regulation of gene expression	[40]
***Ppp1r1b*** *	h, m	Striated muscle differentiation	PRC2 interaction	[41]
***Charme*** *	h, m	Striated muscle differentiation	Chromatin interaction	[42]
*MIAT ^*	h	SNP increases MI risk	?	[54]
*Mhrt ^*	m	Cardioprotective	Modulation of chromatin	[50]
*LIPCAR ^*	h	Cardiac remodeling	?	[55]
*ANRIL ^*	h	Cardiac event biomarker	?	[56]
*KCNQ1OT1 ^*	h	Cardiac event biomarker	?	[56]
***Malat1 ^***	h	Cardiac event biomarker	?	[56]
*CARL ^*	m	Suppress mitochondrial fission	miR-539 sponge	[57]
*CRP*	m	Cardiomyocyte proliferation	Promotes methylation	[49]
*CRLL*	r	Cardiomyocyte proliferation	miR-199a3 sponge	[58]
***H19*** *^	h, m	Cardiac development	Cardiomyocyte differentiation and hypertrophy	[31,52]
***Meg3 ^***	h, m	Cardiac fibrosis	P53 interaction	[53,59]
***Caren ^***	m	Cardioprotective	Decrease Hint1 translation	[51]
*LncDACH1 ^*	h, m	Cardiomyocyte proliferation	PP1A inhibitor	[48]
*CAREL*	h, m	Cardiomyocyte proliferation	ceRNA for miR-296	[47]
*CARDINAL ^*	h, m	Cardioprotective	Inhibits SRF/TCF complex	[60]

* indicates lncRNAs which are specific to development; ^ indicates lncRNAs implicated in disease; **Bolded** text denotes those lncRNAs which are in both cardiac and skeletal muscle. Key: m = mouse, h = human, p = primate (human and non-human), and r = rat.

**Table 2:** Summary of key lncRNAs found in skeletal muscle.

Name	Species	Function	Mechanism	Citation
Evx1as *	m	Skeletal muscle differentiation	Chromatin binding	[69]
yylncT *	h	Mesodermal lineage commitment	Binds to DNMT3B	[70]
Sirt1 AS *	m	Promotes proliferation and represses differentiation	Sirt1 mRNA binding	[90]
Syisl *	m	Repress differentiation	Recruits Ezh2	[91]
*LncMyoD*	h, m	Promotes differentiation	Blocks IMP mediate translation	[72,73]
*linc-MD ^*	h, m	Promotes muscle specific transcription factors and differentiation	Sponges miR-133 and miR-133	[74,76]
*lnc-mg*	m	Promotes differentiation	Sponges miR-125b	[77]
*lnc-YY1*	h, m	Promotes differentiation	Binds YY1	[78]
*MUNC*	m	Gene expression regulation	MyoD binding	[79]
***Malat1***	h, m	Proliferation and differentiation	Recruitment of Suv39h	[81,82]
*Dum*	m	Promyogenic	Silences Duppa	[80]
***Meg3***	h, m	Myoblast differentiation; Regulates EMT	PRC2 interaction	[85]
*m1/2-sbsRNA*	m	Promote cell activation	mRNA degradation	[71,86]
*Yam-1*	m	Repress differentiation	Activates miR-715	[87]
*lnc-31 ^*	h, m	Promote proliferation and inhibits differentiation	?	[89]
*Chronos*	m	Inhibition of hypertrophic growth	Represses Bmp7	[92]
*SRA*	h, m	Regulation of gene expression	Ribonuclear complexes	[96]
*DBE-T ^*	h	De-repression of 4q35 genes	Interaction with Ash1L	[94]
*circ-ZNF609*	h, m	Proliferation	?	[99]
*LINC00961*	h, m	Micropeptide	mTOR mediated regeneration	[95]
*Myoregulin*	h, m	Micropeptide	Impedes calcium reuptake	[14]
*DWORF*	m	Micropeptide	Enhances SERCA activity	[100]
***H19 ^***	h, m	Promotes myoblast differentiation/ Stabilizes dystrophin	Mediated via miR-675-3p and miR-675-3b; Association with dystrophin	[83,84,93]
Ppp1r1b *	h, m	Striated muscle differentiation	PRC2 interaction	[41]
Charme *	h, m	Striated muscle differentiation	Chromatin interaction	[42]

* indicates lncRNAs which are specific to development; ^ indicates lncRNAs implicated in disease; **Bolded** text denoted those lncRNAs which are in both cardiac and skeletal muscle. Key: m = mouse and h = human.

## Data Availability

Not applicable.
